# From metabolic reprogramming to epigenetic modification: association network and targeted treatment strategy between histone lactylation and tumor progression

**DOI:** 10.3389/fimmu.2025.1542664

**Published:** 2025-09-17

**Authors:** Yi Li, Lu Feng, Qian Shen, Xiaochen Jiang, Fudong Liu, Chuanlong Zhang, Runzhi Qi, Bo Pang

**Affiliations:** ^1^ Guang’anmen Hospital, China Academy of Chinese Medical Sciences, Beijing, China; ^2^ First Clinical Medical College, Shandong University of Traditional Chinese Medicine, Jinan, China; ^3^ Beijing Chao-yang Hospital, Capital Medical University, Beijing, China; ^4^ Department of Traditional Chinese Medicine, Beijing Friendship Hospital, Capital Medical University, Beijing, China; ^5^ Xiyuan Hospital, China Academy of Chinese Medical Sciences, Beijing, China

**Keywords:** metabolic reprogramming, epigenetic modification, histone lactylation, tumor, targeted therapy

## Abstract

Metabolic reprogramming and epigenetic modification have been widely observed in cancer research. Based on accumulating experimental evidence in recent years, beginning with metabolic reprogramming driven by carcinogenic signals, the accumulation of key metabolites, represented by lactate, continuously affects cellular plasticity and alters the epigenetic landscape. As a new post-translational modification of histone, histone lactylation not only changes the nucleosome structure, but also regulates chromatin dynamics and gene expression, which is closely related to the poor prognosis of tumors, contributing to immune escape, immune monitoring and angiogenic events in tumor progression. Before the discovery of histone lactylation in 2019, there was a lack of systematic understanding of the lactate regulation of tumor metabolism, immune effects and microenvironmental homeostasis. From metabolic changes to stable gene expression, histone lactylation has become an important entry point in tumor research, connecting the relationship network of metabolic reprogramming, Tumor microenvironment (TME) and epigenetic modification. It represents an important conceptual link between metabolism and epigenetics, and emerging evidence suggests it may be a promising area for understanding tumor progression and developing targeted therapies. In this review, we focus on how tumor cell metabolic reprogramming reshapes the epigenetic landscape into histone lactylation. Besides, we discussed the plasticity of tumor metabolism regulated by histone lactylation in reverse, involving TME biological processes such as immunity and metabolism. Finally, we reviewed the new molecular targets and targeted therapeutic strategies of histone lactylation for cancer treatment. Elucidating these problems will provide theoretical basis for further research and clinical application in this field in the future.

## 1 Introduction

Lactate is linked to the glycolytic pathway (GP) and oxidative phosphorylation, and is considered as an end product of glucose oxidative metabolism and an intermediate product of GP process in classical theory of energy and matter metabolism. As an important oxidizing substrate in the aerobic energy supply system involved in energy metabolism (1), lactate has been regarded as a metabolic waste and fatigue agent in the past. However, more and more studies have helped us to realize that lactate is an important messenger in the complex feedback loop system of biological metabolic process. As a key signaling molecule, lactate not only participates in the lactation of allosteric binding and lysine residues, but also regulates a variety of extracellular and intracellular biological and kinetic activities, including immune cell metabolism, TME and gene expression (2, 3). As a ‘metabolic waste’, the shift in lactate status stems from the discovery of the Warburg effect, a metabolic signature that reveals how tumor cells preferentially consume glucose to produce lactate through GP in the event of metabolic disorders (4). In recent years, this metabolic pattern centered on lactate formation has also been found to promote the proliferation of tumorigenic immune cells, and contribute to immune escape, immune monitoring, and angiogenic events in tumor progression (5).

The epigenome refers to the dynamic heritable chemical changes in the genome that occur independently of the DNA sequence, and these modifications can affect gene expression. Protein posttranslational modifications (PTM) are modifications that add or remove specific chemical groups to one or more amino acid residues on the translated protein. This covalent modification can regulate protein activity, localization, folding state, and interaction with other biological macromolecules (6). Cellular metabolism has been shown to be an important modifier of various epigenetic modifications, such as histone lactylation, methylation and crotonylation, and DNA methylation (7). A study by Zhang et al (8). shows that lactate can change the epigenetic landscape. Through lactation, histone lysine residues promote epigenetic regulation of genes, directly stimulating gene transcription of chromatin in T cells and influencing the future fate of the cell. And through covalent modification, amino acid residues on histones can link different acyl groups. The modification disorder of histone lactylation alters the structure of nucleosomes and disrupts the balance of gene transcription. These tiny errors are amplified during gene expression, affecting chromatin dynamics, and then gene silencing or transcriptional activation (9). Yang et al. extensively observed histone lactylation in macrophages and found that lactate could induce lactation of high mobility group box-1 (HMGB1) (10). Meanwhile, lactate also stimulated the acetylation of HMGB-1 through Hippo/YAP-mediated Sirtuin 1 (SIRT 1) and β-arrestin 2-mediated p300/CBP recruitment, promoting the release of HMGB-1 from macrophages. Thus, histone lactylation causes a variety of biological changes that regulate tumor progression, inflammation, and immune responses (11, 12).

Metabolic reprogramming and epigenetic changes have been widely observed in cancer and are seen as important factors contributing to cancer progression. Changes in the ways and processes of metabolism of primary fuels such as sugars, amino acids and fats in tumor cells are usually caused by epigenetic changes. As a measure of lactate level, histone lactylation is closely related to cell metabolism. At the same time, it drives the association network between metabolic reprogramming and tumor, regulating the metabolic plasticity of tumor cells (13, 14). Teasing out these relationships, we found that tumor cells can be widely affected by lactation. As a key part of this process, the accumulation of lactate not only affects histone lactylation, regulates metabolic homeostasis, but also enters endothelial cells through monocarboxylate transporter, stimulates NF-κB/interleukin (IL)-8 pathway, and induces angiogenesis and cell migration (15, 16). Unlike mutations, epigenetic changes are reversible, which offers possible new options for cancer treatment research. Therefore, it is reasonable to conclude that lactation-induced lactation of histone lysine residues deserves research attention. This epigenetic modification from lactate effectively promotes transcriptional dependence and accelerates the malignant development of tumor cells, becoming an important factor in the poor prognosis of tumor patients.

In this study, we focused on how tumor cell metabolic reprogramming reshapes the epigenetic landscape into generate histone lactylation, comprehensively reviewing and summarizing the available evidence on tumor lactate metabolism and histone lactylation driving metabolic reprogramming in reverse. We summarized in detail that lactate regulates tumor metabolism and microenvironment, and histone lactylation promotes tumor progression. At the same time, focusing on the new perspective of histone lactylation, we review new options for tumor targeted therapy and summarize the recognized challenges and future prospects of this promising area of research. Based on this systematic and comprehensive framework, we expect to reconstruct the overall landscape of the impact of histone lactylation on tumor progression from multiple perspectives, providing a basis for further research and clinical application of histone lactylation.

## 2 Warburg effect and tumor lactate metabolism

### 2.1 Impaired glucose intake and the Warburg effect

The energy supply of the organism is indispensable to the life activities of the organism, and glucose is the main energy source of cell proliferation, of which the metabolic process is the main way for cells to produce energy and biosynthetic materials. In normal life, Cellular glucose uptake is regulated by a variety of signals, which often from the epidermal growth factor (EGF), platelet-derived growth factor (PDGF), and insulin stimulation (17). There are two main types of glucose metabolism, one is GP, pentose phosphate pathway (PPP) and serine synthesis pathway (SSP) in the cytoplasm, and the other is oxidative phosphorylation in mitochondria (18). In normal tissues without mutation, 90% of the energy required comes from the energy metabolism mode of mitochondrial oxidative phosphorylation, and the energy synthesized by GP only accounts for 10% of the total supply. GP is a common process in glucose metabolism, one of the results of which is the production of pyruvate. Being converted to lactate and released outside the cell, or into the mitochondria, pyruvate participates in the depletion of the tricarboxylic acid cycle. In addition, GP intermediates can also enter PPP and SSP (19).

At the beginning of the 20th century, German physiologist Warburg observed that tumor cells do not use oxidation as the main energy supply mode even when oxygen supply is sufficient, but cooperate with glucose consumption to produce lactate with low efficiency GP to complete energy supply, which is called Warburg effect (20). The Warburg effect is regarded as a typical feature of metabolic reprogramming and is widespread in many tumors such as lung cancer, breast cancer and colorectal cancer (21–23). Hypoxia induces metabolic changes in tumor cells to better adapt to deprived nutrient consumption patterns. Enhanced glycolysis is the most important metabolic marker during hypoxia. Dysregulation of glucose uptake stimulated the proliferation of tumor cells and facilitated the entry of a relatively small portion of the carbon source into the mitochondria (24). Why would a tumor cell choose to metabolize in a way that is so inconceivable from a common sense perspective? In fact, this energy metabolism, although very inefficient, is very advantageous for tumor cell metabolism. Compared with oxidative phosphorylation, GP productivity pathway is simple, and the energy metabolism process is time-saving. At the same time, GP provides intermediate metabolites for tumor proliferation and reduces reactive oxygen species levels to a certain extent (25, 26). In recent decades, it has been found that the energy selective migration of tumor cells is regulated by their own genes and related to microenvironment changes, which is the result of selective inhibition of mitochondrial oxidative phosphorylation (27, 28). Although the hypothesis proposed by Warburg has been challenged by new research results in recent years, it is undeniable that different degrees of mitochondrial function damage do exist in some tumor cells (29).

### 2.2 Formation, transport and consumption of lactate in tumor cells

#### 2.2.1 Lactate formation

**Figure 1:**
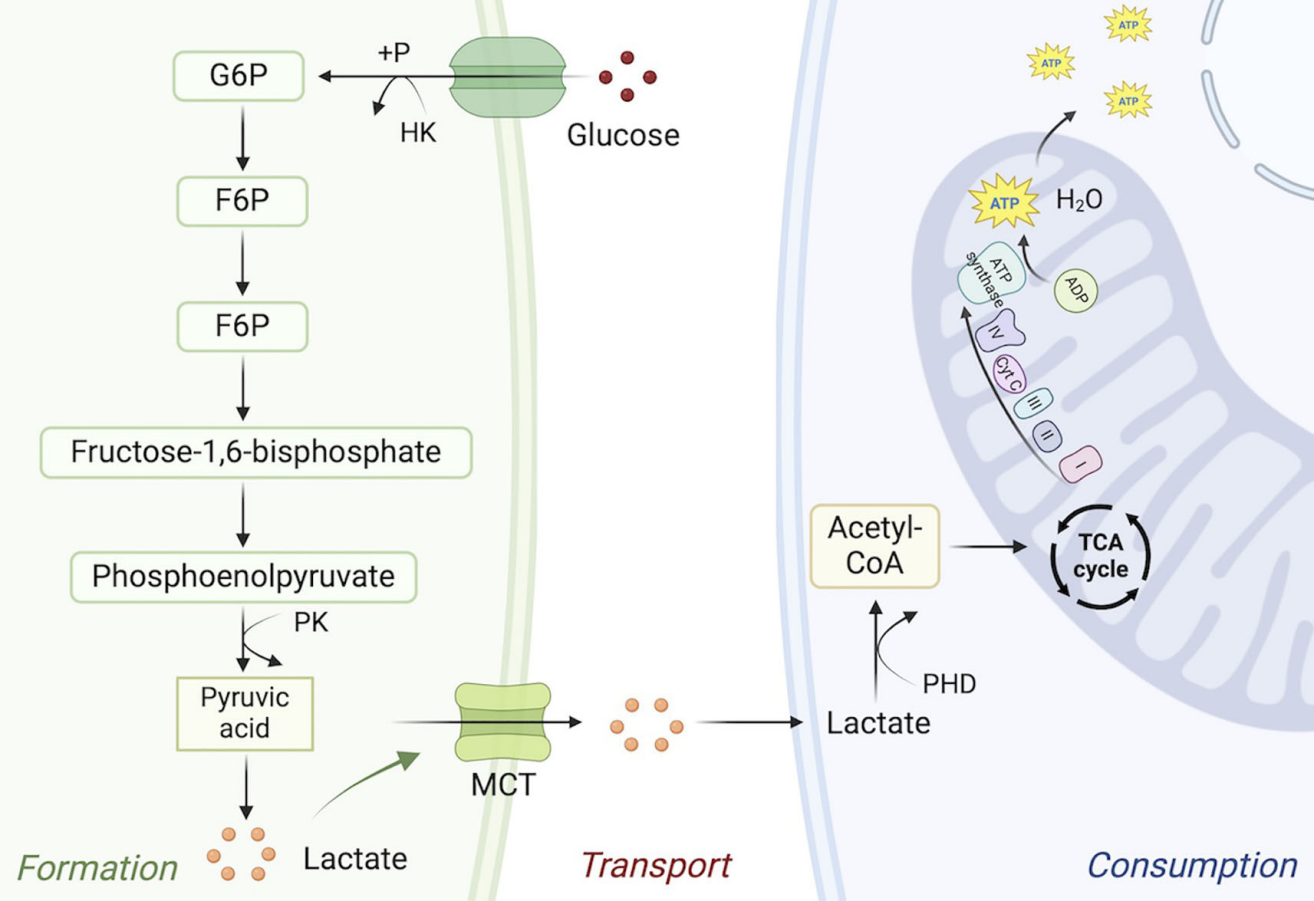
The formation, transport and consumption processes of lactate. Glucose produces lactate through the glycolytic pathway, which is transported extracellular by monocarboxylate transporter and consumed in mitochondria by pyruvate dehydrogenase. +P, phosphorylation; HK, hexokinase; F6P, D-Fructose 6-phosphate; PK, pyruvate kinase.

Lactate is an important substrate for histone lactylation and an inevitable product of GP. In fact, lactate is mainly formed through two modes of GP and glutamine metabolism. GP is a series of enzymatic processes involved. Firstly, glucose transporter transports glucose to cells, and hexokinase catalyzes the phosphorylation of sugars to form glucose-6-phosphate (G6P), which activates glucose, facilitating its further participation in synthesis and catabolism. Subsequently, phosphofructokinase 1 catalyzes irreversible phosphorylation of various sugars to form fructose-1,6-bisphosphate. Finally, the pyruvate kinase converts phosphoenolpyruvate to pyruvate (9). When oxygen supply is insufficient, pyruvate cannot be further REDOX to form lactate. Tumor cells regulate metabolic processes that drive the conversion of pyruvate into lactate, resulting in a large amount of lactate accumulation. Although 1 molecule of glucose in GP can only produce 2 ATP, the energy metabolic path of GP is shorter and the metabolic rate is higher than that of oxidative phosphorylation. Up-regulation of GP energy supply ratio can provide sufficient energy support for tumor cell proliferation and metabolism in the later stage. GP can convert intermediate products of energy metabolism into ribose 5-phosphate (R5P) and NADPH through PPP. The former can participate in the synthesis of lipids and nucleic acids and other substances, while the latter participates in nucleotide metabolism in the form of electron donors, providing synthetic precursor substances for the subsequent growth of tumors (30). Lactate accumulation provides transitional adaptation to aggressive phenotypic transformation of tumors. It has also been reported that lactating MRE11, a key protein of DNA repair, promotes homologous recombination repair, affecting subsequent therapeutic resistance (31).The formation, transport and consumption processes of lactate are shown in **Figure 1**.

#### 2.2.2 Lactate transport

With the further study of lactate metabolism, we have a more comprehensive understanding of the process of lactate transport. lactate can be produced by most tissues of the body, the concentration of lactate in human serum is 1–3 mM at rest, 15mM at intense exercise, and the level of lactate in tumors can be as high as 40 mM (32). Lactate is the metabolic fuel of tumor cells. During GP, lactate formed by cell metabolism is transported extracellular by Monocarboxylate transporters (MCT), preventing intracellular accumulation leading to negative feedback inhibition of GP flux. MCT is a transmembrane transporter with 14 subtypes in its family, which has the powerful ability to transport lactate, pyruvate, ketone bodies, thyroid hormones and aromatic amino acids (33). The proton-dependent transporter MCT1–4 is mainly responsible for the transport of lactate, but unfortunately, no more structural reports on MCT have been found in the studies on lactate transport. Research on MCT often focuses on the way of functional simulation, while there are few studies on MCT Whether MCT5 can transport lactate is also a problem that needs to be studied in the future, making the specific mechanism of lactate transport more mysterious (34). In the case of carbohydrate deprivation, Duan et al. (35) found that the expression rates of MCT1 and MCT4 were significantly increased, and lactate could maintain a high productivity level and resist cell death of U251 cells. It was also found that lactate can induce lactate transport and convert the dominant Warburg effect into oxidative phosphorylation. To a certain extent, lactate transport is also regarded as a form of metabolic symbiosis between tumor cells. Lactate transport is stimulated by the transmembrane concentration gradient, and tumor cells create a pseudo-hypoxic environment by generating fibroblasts based on hydrogen peroxide, further activating the expression of MCT 4 and promotes the output of lactate from stromal cells, which is then absorbed by cancer cells and used for proliferation (36, 37). This form of lactate transport, driven by tumor cells, constitutes the transport framework for lactate to shuttle within the tumor, regulating lactate homeostasis (38).

#### 2.2.3 Lactate consumption

Excessive lactate accumulation is dangerous and can lead to a condition called lactate acidosis. Therefore, the body has evolved a set of rapid metabolic mechanisms of lactate clearance and consumption to maintain lactate homeostasis. In TME, hypoxic cell populations produce large amounts of lactate through the Warburg effect. Lactate is transported to adjacent normal oxygen-supplying cells through MCT, and oxidative phosphorylation occurs, consuming lactate to produce energy. Lactate is irreversibility consumed by Pyruvate dehydrogenase (PDH), entered the tricarboxylic acid cycle by using acetyl-Coenzyme A as raw material and metabolized by kidney and liver. The horizontal relationship between PDH and GP flux is a potential influence on lactate consumption. As a component of the catalytically active complex, PDH is inhibited by E1α subunit and reduced coenzyme I to regulate the increase of circulating lactate level (39, 40). Lindsay et al. (41) reported that changes in cellular REDOX status and mitochondrial function can affect metabolic coupling. The expression level of catabolic transcription factors such as Hypoxia inducible factor 1α (HIF1α) was negatively correlated with oxidative phosphorylation metabolism and was highly expressed in tumor-associated fibroblasts. These studies suggest that different metabolic phenotypes of tumor cells regulate McT-mediated lactate transport processes, thereby affecting lactate consumption networks.

### 2.3 Lactate regulates tumor metabolism and microenvironment

With the deepening of lactate research in recent years, researchers have increasingly found that lactate plays an important role in the progression of tumor diseases, rather than just the role of metabolic waste as previously thought (42). In reprogrammed TME, lactate establishes a complex coupling between metabolic and genetic variation, linking metabolism, inflammation, and cancer (43). In terms of metabolism, lactate can regulate the expression of metabolic genes and promote the growth of tumors. G-protein-coupled receptor (GPR)81 has been found in patients with a variety of tumors, including breast and colon cancer (44, 45). Studies have shown that lactate can act as a signaling molecule, activate the GPR81 pathway, regulate the expression of metabolism-related genes, promote tumor angiogenesis, inflammatory damage, affecting immune escape events (46, 47). Liu et al. found that lactate was the highest heterometabolite in unbiased metabolomic screening of tumor cachexia mouse models (48). Ablation of GPR81 in the host has no effect on tumor load, while its defects in cancer cells hinder tumor growth. Lactate can significantly stimulate GPR81, and the experiment also confirmed that lactate can promote the fat metabolism remodeling process of tumor progression through the Gαi/o-Gβγ-RhoA/ROCK 1-p38 signaling cascade. Interestingly, the activation of GPR81 by lactate to regulate tumor metabolism has also been found in lung cancer. In lung cancer cells, inhibition of GPR81 signaling leads to decreased levels of the PD-L1 protein and disruption of the active function of the PD-L1 promoter. Feng et al (49). revealed the unexpected role of lactate in promoting tumor cell protection from cytotoxic T cell targeting. These results indicated that the up-regulation of PD-L1 expression in lung cancer mainly plays a role in lactate-rich TME, which induces tumor cells to activate PD-L1, resulting in reduced production of interferon-γ, and inducing apoptosis of Jurkat T-cell leukemia cells cultured together. Interestingly, lactate also regulates the expression of genes associated with lipid metabolism in prostate cancer cells, establishing a regulatory ring between metabolites and epigenetic modifications and promoting prostate cancer metastasis (50).

TME is an acidic, inflammatory and hypoxic cellular environment composed of tumor cells, tumor-infiltrating immune cells, tumor-associated fibroblasts, glial cells, endothelial cells, immune factors and cytokines (51). The up-regulated GP metabolic pattern generates a large amount of lactate in the production process, resulting in microenvironment acidification, which inhibits the ability of immune system to recognize and resist tumors (52). As a metabolic fuel and signaling molecule, lactate directly affects many cellular activities in TME, and directly or indirectly participates in the inhibition of immune cell function in TME (53). Lactate induces and recruits immunosuppression-related cells and molecules in TME, activates intracellular signaling pathways, and regulates cell behavior, exhibiting complex immunosuppressive functions. At the same time, studies have also shown that, combined with the modification and transformation of inflammatory immune cell phenotypes and the delivery of inflammatory immune factors, excessive lactate accumulation can up-regulate cell aggregation and migration, induce tumor invasion and metastasis, and accelerate the occurrence of immune escape.

Lactate also significantly affects the expression of PD-1 and effector T cells, leading to immune escape and increasing the difficulty of chemotherapy treatment (54). Neutralizing the acidic tumor environment is considered a strategy to enhance immunotherapy resistance to tumors. For example, acidosis can cause tumor-specific CD8^+^ T cells in humans and mice to enter a non-functional state, and after treatment with proton pump inhibitors, immune function reversal occurs in some T cells (55). Quantization of glucose uptake in activated CD8^+^ T cells using the fluorescent glucose analogue 2-NBDG, Sukumar et al. It was found that increasing lactate levels promoted CD8^+^ T cells to a state of terminal differentiation. However, the activation of CD8^+^ T cells by GP inhibitor 2-deoxyglucose significantly enhanced the generation of memory cells and anti-tumor ability (56). Lactate formation is accompanied by a lack of oxygen in the microenvironment. Tumors that occur on the mucosal surface, when cells proliferate away from the substrate source, can also lead to hypoxia. In the hypoxic microenvironment, by changing the metabolic pattern, tumor cells can inhibit the anti-tumor effect of immune cells and trigger the induction mechanism of the microenvironment, which leads to the transformation of inflammation into cancer (57).

## 3 Histone lactylation drives metabolic reprogramming and cancer association networks

### 3.1 Discovery and advanced understanding of histone lactylation

**Figure 2:**
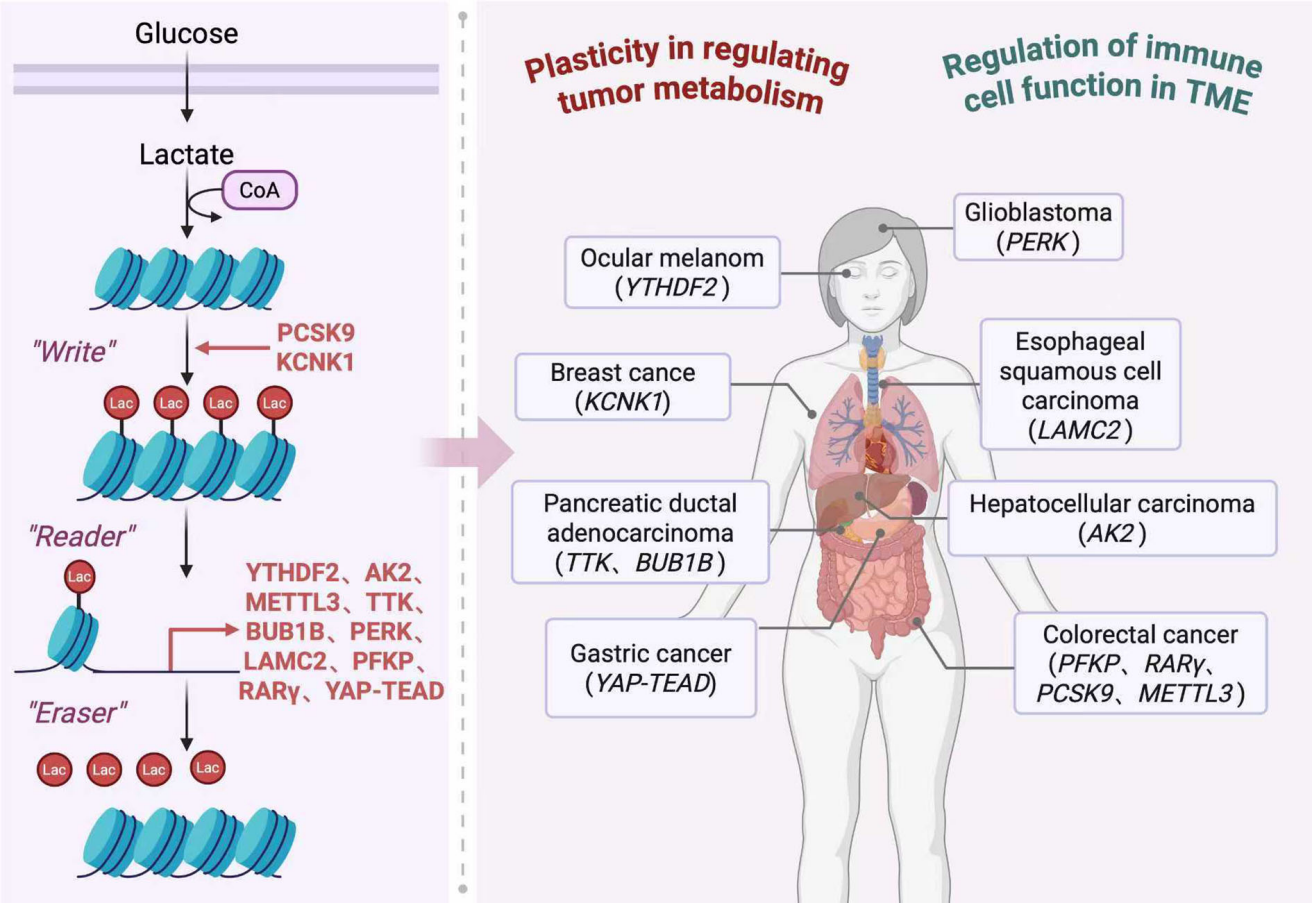
The process of histone lactylation and the mechanism of its action on tumor. In the process of histone lactation, “Writers” refer to proteins responsible for adding lactating modification groups to histones, “Readers” refer to proteins capable of recognizing and binding specific lactating modification groups, and “Erasers” refer to proteins responsible for removing lactating modification groups from histones. PCSK9 can affect the level of histone lactation modification by regulating cellular lactate metabolism and the activity of histone lactation modification enzymes. KCNK1 can promote histone lactation by facilitating cellular glycolysis and lactic acid production. A variety of proteins affect tumor progression through active or passive participation in histone lactylation.

Epigenetic modification includes histone modification and DNA modification, which can regulate a series of downstream effects by activating or silencing genes. Therefore, in recent years, epigenetic modification has been regarded as an important entry point for the treatment of diseases, especially in the study of tumors (58, 59). As mentioned earlier, the role of lactate as a carcinogenic signaling catalyst is well established. It is worth noting that lactation is a novel post-translational modification that involves covalent linking of lactate to protein lysine residues, directly stimulating or inhibiting gene transcription of chromatin. More and more evidence confirms the key mechanism of lactate in helping to alter histone lysine residues. This dynamic and reversible epigenetic modification was first reported by Zhang et al.’s team (8). The presence of histone lactylation was confirmed by mass spectrometry mass transfer matched to the lactate group and western blotting of pan-anti-lactating antibodies. The researchers found that lysine residues increased in a dose-dependent manner as the amount of lactate increased. At the same time, this study confirmed that lactate activates M2-like gene expression through histone lactylation, that histone lactylation is regulated by GP, and that histone lactylation is induced by hypoxia. It is worth emphasizing that so far, the powerful effects of histone lactylation are far beyond our imagination, such as regulating the immune environment, affecting tumor metabolic reprogramming, regulating aging, affecting gene expression regulation, participating in cell signaling, and even changing cell fate (60–62). Histone lactylation and its effects on tumors are shown in **Figure 2**.

There is growing evidence that lactate interferes with tumor progression in a variety of ways. It not only comes from direct metabolic pathways, but also plays a key role in tumor progression through complex mechanism networks through epigenetic modification. In fact, a number of findings in recent years seem to tell us that histone lactylation has important research significance for the research of inflammatory diseases, cardiovascular diseases, nervous system diseases and other diseases, rather than just confined to the tumor research level (63). At the inflammatory level, as mentioned earlier, histone lactylation is associated with the repolarization of the M1 to M2 phenotype, such as driving the transformation of pro-inflammatory M1 macrophages into anti-inflammatory M2 macrophages. Under inflammatory conditions, M1 macrophages exhibit high GP activity, leading to the production of large amounts of lactate. In contrast, M2 macrophages have strong tissue repair ability, mainly releasing anti-inflammatory cytokines, and can synthesize proteins such as collagen, contributing to the repair of damaged tissues (9). Combined with other evidence, macrophages with fragmented mitochondrial phenotypes promote mitochondrial fragmentation leading to increased cellular lactate. Inflammatory damage drives epigenetic changes, and histone lactylation occurs simultaneously with IL-6 and ARG-1-dependent metabolic rewiring under inflammation, causing an increase in mitochondrial enzyme 1 expression and promoting the development of inflammation towards regression (64, 65). During the anti-inflammatory process after myocardial infarction, histone lactylation modulates the dual anti-inflammatory and angiogenic activity of monocyte macrophages by promoting the transcription of restorative genes such as LRG 1, VEGF-α and IL-10 (66).

### 3.2 Comparison of histone lactylation with other modifications

Histones can undergo a wide range of PTM, and the epigenome refers to heritable, dynamic chemical changes in the genome that occur independent of DNA sequences. Histone modification refers to A series of modifications on the amino acid residues of histones, which can include a variety of types, such as lactate is responsible for the lactation of histones, metabolism-related enzymes participate in the regulation of histone phosphorylation, and acetyl-CoA affects the acetylation of histones (67). The differences between histone lactylation and other modifications are mainly reflected in the difference of regulatory enzymes and functional differences. For example, the regulatory enzymes of histone acetylation are histone acetyltransferase and histone deacetylase. Acetylation is usually associated with the activation and expression of genes, such as the regulation of cell cycle and cell proliferation and apoptosis. By weakening their interaction with negatively charged DNA, acetylation neutralizes the positive charge on the lysine amine group, substantially increasing its hydrophobic properties, reducing the lysine side chain’s ability to form hydrogen bonds (68). The main regulatory enzymes of histone phosphorylation are protein kinase and protein phosphatase. In terms of function, it can usually participate in the interaction between histone and DNA, and the regulation chromatin remodeling and gene expression, such as phosphorylation of H3S10 is related to the transcriptional activation of chromatin. Meanwhile, phosphorylation can regulate the selective access of methylated lysine, which can indirectly protect the epigenetic process of cell division (69, 70). From the perspective of recognizing modified histones, differentiated PTMS control different chromatin dynamics and specific transcriptional profiles (71). There are also interactions between different chemical modifications, so somatic reprogramming provides us with a solution. Li et al. (72) found that the transcription factor Glist induced multi-level epigenetic and metabolic remodeling in stem cells, droving the coordination of histone acetylation and lactation in GP, and forming the epigenome-metabolome-epigenome signal cascade network. The same examples, such as lactation and acetylation modification, are regulated by HATs and HDACs, and even in some tumor cells, there is an increase in lactation level and a decrease in acetylation level, which is related to potential competition for common substrates (11, 14).

### 3.3 Histone lactylation regulates the plasticity of tumor metabolism

The occurrence of tumor diseases is related to the disruption of the balance of gene transcription by the modification of histone lactylation. A study conducted by Li et al. (73) showed that patients with metastatic colorectal cancer who were resistant to bevacizumab would have elevated histone lactylation levels, while inhibiting the histone lactylation process could effectively inhibit the progression of metastatic colorectal cancer and improve the prognosis of patients. Yu et al. (60) first revealed the carcinogenic effect of histone lactylation and its adverse effect on patient prognosis. It was found that YTH N6-methyladenosine RNA-binding protein 2 (YTHDF2) could recognize and promote the degradation of M6A-modified PER 1 and TP 53 mRNA, and histone lactylation affected the overexpression of YTHDF2 and promoted the occurrence of tumors. Another study on liver cancer collected a global lactate group analysis of a hepatitis B virus-associated hepatocellular carcinoma cohort, identifying the lactate sites of 9,275 tumors and adjacent livers. The study also found that lactation of K28 inhibits adenylate kinase 2. Affecting the metabolism of sugars, amino acids, fatty acids and nucleotides, lactation also shows extensive effects on a variety of metabolism-related enzymes. It indicated that histone lactylation plays an important role in regulating cellular metabolic plasticity, potentially promoting the development of liver cancer (74).

Compared to previous studies, with the help of liquid chromatography-tandem mass spectrometry, Song et al. (75) found 1011 lactating sites in 532 proteins and 1197 lactating sites in 608 proteins in SCC25 cells. It is proved that histone lactylation is only a part of lactated proteins, and there are also lactic modification phenomena in non-histone proteins. It should be noted that this discovery deepens our comprehensive understanding of histone lactylation, and also enhances the important position of systemic lysine-lactate substrate in the whole regulation process. Just as a coin has two sides, the regulatory mechanism of chemical modification is not completely absolute. In the cellular metabolism of non-small cell lung cancer, Jiang et al. (76) observed that lactate down-regulates and up-regulates mRNA levels of glycolytic enzyme and TCA circulating enzyme. This results in the inhibition of glucose uptake and GP of tumor cells, as well as the reduction of cell proliferation and migration, producing a certain anti-cancer effect. This also happens in uveal melanoma. Longhitano et al. (77) found that histone lactylation can increase cell homozygote by modifying H3K18la, resulting in a decrease in proliferation and migration of uveal melanoma cells, further inhibiting the progression of uveal melanoma. Although the dual function of histone lactylation is very interesting, from the perspective of the specific mechanism of tumor, histone lactylation is still labeled as promoting tumor progression in the vast majority of tumors. As the current hot research direction, it is very necessary to continue and in-depth exploration of histone lactylation.

### 3.4 Histone lactylation regulates immune cell function in TME

Immune cells play an important role in TME, participating in anti-tumor immune response, immune surveillance, immune regulation and immune escape. Combined with the existing research evidence, istone lactylation can regulate the metabolic process of immune cells in TME, inhibiting the proliferation of natural killer cells, dendritic cells, and CD8+ T cells, and mediating immune escape events (78). Deng et al. evaluated the effect of lysine lactylation on tumor immunity and found that oncogenes such as NDUFAF6, OVOL1 and SDC1 were negatively correlated with activated NK cells in breast cancer. While lysine lactylation was positively correlated with macrophage M2, indicating that lysine lactylation greatly affected the function of immune cells in TME (79). Originating from monocytes, macrophages are key types of cells in the immune system, mainly clearing dead cells, regulating inflammation and participating in tissue repair (80). As the first responders of the immune system, macrophages can be polarized into the pro-inflammatory, anti-cancer M1 type and the anti-inflammatory, pro-cancer M2 type. Macrophages use tumor-derived lactate in TME to promote the tumor phenotype through histone lactylation (81). Wang et al (82). used colon cancer tissues to analyze the association between proprotein convertase subtilisin/kexin type 9 (PCSK9) expression and clinicopathological factors of patients. Studies have shown that the progression of colon cancer is related to the epithelial mesenchymal transformation of tumor cells regulated by PCSK9 and PI3K/AKT signaling, which regulates the level of histone lactylation, playing a role in phenotypic polarization of macrophages. Tumor infiltrating myeloid cells (TIMs), including macrophages, refer to a class of immune cells from bone marrow that are widely involved in tumor immune escape. A study exploring lactation and TME showed that lactate accumulation in TME can effectively induce upregulation of methyltransferase-like 3 (METTL3) in TIMs through H3K18 lactation, which is associated with poor prognosis of colorectal cancer (83). In a strict sense, the regulation of tumor progression by histone lactylation is not limited to regulating the cell cycle of tumor cells, the expression of genes related to DNA repair and metabolic reprogramming, and inhibiting the activity of immune cells in TME. The detailed mechanism of histone lactylation’s involvement in tumor regulation is shown in **Table 1**.

**Table 1:** The mechanism of histone lactylation involved in the regulation of tumor.

Cancer	Cell line/animal model/patient specimens	Lactylation sites	Mechanism and effect	Ref
Ocular melanoma	OCM1 cells; CRMM1 cells; 92.1 cells; MUM2B cells; MEL290 cells; OMM1 cells; CRMM1 cells; CRMM2 cells; CM2005.1 cells; male BALB/c nude mice (4 weeks old); human ocular melanoma tissues; human normal melanocyte tissues	Pan-lysine lactylation; H3K18 la	Histone lactylation promotes cancer by the m^6^A reader YTHDF2, finding that PER 1 and TP53 may be key candidate genes for YTHDF 2.	(60)
Colorectal cancer	FHC cells; SW480 cells; SW620 cells; HCT 116 cells	Pan-lysine lactylation; H4K12 la; H3K14 la; H4K8 la	FHC cells use the negative feedback mechanism to involve the lactic acidification-dependent inhibition of the speed-limiting enzyme PFKP in GP. The PFKP lactication in SW480 cells and colon cancer tissue directly attenuates the activity of the enzyme. The distuned lactic acid/la-PFKP negative feedback ring plays an important role in the progression of colon cancer.	(84)
	HEK293T cells; SW480 cells; HCT116 cells; THP-1 cells; RAW 264.7 cells; MC38 cells; C57BL/6 mice (6–12 weeks old); BALB/c nude strain mice (6–7 weeks old); human primary colorectal cancer and corresponding nontumor tissues	H3K18 la	The downregulation of the expression of RARγ in macrophages is related to the poor prognosis of colorectal cancer patients. Lactic acid from tumors promotes H3K18 lactation thereby inhibiting the transcription of the RARγ gene in macrophages, increasing the level of IL-6, and promoting the tumor progression of macrophages by activating signal transductors and transcription activator 3 (STAT 3) signal transduction in CRC cells.	(85)
Pancreatic ductal adenocarcinoma	hTERT-HPNE cells; MIA PaCa-2 cells; PANC-1 cells; AsPC-1 cells; PL45 cells; female BALB/c nude mice (4–5 weeks old); pancreatic samples of pancreatic ductal adenocarcinoma and paired paracancerous controls; serum samples from patients with pancreatic ductal adenocarcinoma and healthy controls	Pan-lysine lactylation; H3K18 la	Histone lactylation is associated with poor prognosis in patients with pancreatic ductal adenocarcinoma. Lactate promotes TTK and BUB1B transcription through H3K18 lactate process. TTK and BUB1B in turn promote GP, increase lactate, and promote cell proliferation and migration through continuous positive feedback regulation.	(86)
Glioblastoma	SCC195 cells; GL261 cells; SB28 cells; female C57BL/6 mice (6–8 weeks old); patient peripheral blood and tumor tissue samples	Pan-lysine lactylation	Histone lactylation acidification participates in PERK-driven glucose metabolism and ATF4-regulated GLUT1 expression, regulates mononuclear cell-derived macrophage IL-10 expression, changes immunosuppressive activity, and promotes tumor progression.	(87)
Gastric cancer	HGC27 cells; AGS cells; HEK293FT cells; 293A cells; female BALB/c nude mice (6 weeks old)	Pan-lysine lactylation; H3K18 la	AARS 1 senses intracellular lactic acid and translocates into the nucleus to lactate and activate the YAP-TEAD complex, YAP-TEAD lactylation promotes the expression of Hippo pathway target genes, AARS 1 forms a positive feedback loop with YAP-TEAD to promote gastric cancer cell proliferation.	(88)
Esophageal squamous cell carcinoma	KYSE30 cells; male BALB/c nude mouses (8 weeks old)	H3K9 la	In hypoxia, the level of histone lactylation lactic acidification of esophageal squamous cell cells increases, and histone H3K9la can activate LAMC 2 transcription to promote the progression of esophageal squamous cell carcinoma.	(89)
Breast cancer	MDA-MB-231 cells; MCF-7 cells; female BALB/c nude mice (4 weeks old)	H3K18 la	Potassium channel protein KCNK1 promotes the process of GP and histone lactic acid by binding and activating lactate dehydrogenase A in a non-ionic channel-dependent manner, thereby affecting the expression of downstream genes related to breast cancer cell proliferation, invasion and metastasis, and maintaining the malignant progression of breast cancer.	(90)

## 4 histone lactylation provides a new direction for tumor targeting therapy

The discovery of histone lactylation has become hot point in the field of cancer research. Lactation not only enriches the understanding of tumor pathogenesis and progression, but also has a wide range of clinical application prospects. In particular, with the discovery of new tumor therapeutic targets for histone lactylation-specific genes such as NR6A 1, OSBP 2 and UNC 119B, new drug development has a broader idea (91). At present, the research on tumor histone lactylation still focuses on the main line of inhibiting lactate production. As mentioned above, lactate not only affects histone lactylation, but also regulates TME and metabolism. Therefore, most treatment options revolve around targeting decomposition or synthesis pathways and regulating lactate-related signaling pathways (43). Detailed drug studies targeting histone lactylation are shown in **Table 2**.

**Table 2:** Research on drugs targeting histone lactylation.

Cancer	Cell line/animal model	Pharmaceutical ingredients	Protein modification site	Mechanism and effect	Ref
Liver cancer	HCCLM3 cells; Hep3B cells; female nude mice (6 weeks old)	Demethylzeylasteral	H3K9la; H3K56la	Demethylzeylasteral regulates GP metabolic pathway by reducing lactic acid level in M3 liver cancer stem cells, reduces intracellular lactic acid level in a dose-dependent manner, interferes with metabolic stress-related histone H3 histone lactylation, and inhibits liver cancer stem cell-induced tumor progression	(92)
Hepatocellular carcinoma	Hep3B cells; HCCLM3 cells; nude mice (6 weeks old)	Royal jelly acid	H3K9la; H3K14la	Royal jelly acid reduced the expression of lactic acid in Hep3B and HCCLM3 cells by interfering with GP, inhibited the lactate of H3K9la and H3K14la histone, and then interfered with the production of lactic acid and inhibited the occurrence of liver cancer.	(93)
Prostate Cancer	Human umbilical vein endothelial cells; human mCRPCPC-3 cells; DU145 cells; male BALB/c-nu mice (5 weeks old)	Evodiamine	H3K18 la	Evodiamine, as a metabolic-epigenetic regulator, inhibited histone lactylation and HIF 1α expression in prostate Cancer cells, further enhanced Sema 3A transcription, and inhibited PD-L1 transcription, significantly blocking lactate-induced angiogenesis.	(94)
Glioblastoma	GSC51A cells; 66A cells; SHG141A cells; SHG142A cells; THP-1 cells; BALB/c nude mice (6–8 weeks old)	Oxamate	H3K18la	Oxamate, as an LDHA inhibitor, is able to reprogram glucose metabolism in cancer stem cells, reduce histone H3K18 lactate levels, and inhibit lactate production alter the phenotype of tumor-infiltrating car-t cells in animal models, and mitigate immunosuppression of the tumor microenvironment.	(95)
Metastatic colorectal cancer	SW480 cells; SW620 cells; RKO cells; Caco2 cells; DLD1 cells; Lovo cells; HT29 cells; HCT116 cells; NCM460 cells; HIEC-6 cells; female BALB/c nude mice (6–8 weeks old); tissue specimens in patients with metastatic colorectal cancer	Glycolysis inhibitors	H3K18la	GP-induced histone lactylation drives the progression of metastatic colorectal cancer and promotes the transcription of RUBCNL, which enhances autophagy by promoting autophagosome maturation and maintaining cellular homeostasis; Glycolysis inhibitors can inhibit histone lactylation and enhance the sensitivity of colorectal cancer cells to bevacizumab.	(73)
Pan-cancer	NCI-H460 cells; MCF-7 cells; Hep3Bcells; A375 cells; HT29 cells; LLC cells	PSTMB	None	PSTMB effectively inhibits LDHA activity, targets glucose metabolism, reduces lactate production, and increases induction of apoptotic cell death to reduce tumor cell proliferation.	(96)

### 4.1 Target lactate breakdown or synthesis to reduce histone lactylation

As the direct driving factor of histone lactylation, reducing lactate formation and accelerating lactate decomposition are undoubtedly the first choice for new treatment. Demethylzeylasteral and royal jelly acid have both been shown to reduce intracellular lactate levels in a dose-dependent manner, interfering with the metabolic stress-related histone H3 histone lactylation to inhibit the progression of associated tumors (92, 93). Although current research has confirmed that lactate accumulation is an important factor in tumor progression, when it comes to tumor targeted therapy, this is far from enough, and we must identify the specific mechanisms of this process (97, 98). Some studies have shown that the acidic environment caused by excessive lactate accumulation in TME can negatively regulate tumor-infiltrating immune cells. It affects the crosstalk between tumor cells and neighboring immune cells, forms an immunosuppressive microenvironment, inhibiting immune surveillance function (99–101). In Sun et al.’s study, Oxamate as a lactate dehydrogenase A (LDHA) inhibitor, which has been found to reduce the lactic acidification of histone H3K18, thereby changing the phenotype of immune molecules, promoting the immune activation of tumor-infiltrated CAR-T cells and reducing the immunosuppression of the TME (95). Found in the cytoplasm, mitochondria, and nucleus, LDHA, usually in tetramer form, converts pyruvate to lactate and facilitates the glycolysis process.

LDHA is highly expressed and activated in many tumors, which is related to the malignant progression of tumors (102). In breast cancer, Potassium Two Pore Domain Channel Subfamily K Member 1 has been confirmed to promote tumor cell proliferation and metastasis and accelerate breast cancer progression by activating LDHA and up-regulating H3K18 lactation (90). As a result, drugs targeting LDHA have been evaluated in preclinical trials (103, 104). Kim et al. (96) screened a new LDHA inhibitor, 1-(Phenylseleno)-4-(Trifluoromethyl) Benzene (PSTMB). PSTMB can inhibit LDHA activity and reduce lactate formation without affecting LDHA expression, providing a new option for the development of novel drugs targeting tumor metabolism. Based on the characteristics of tumor metabolic transformation, inhibition of tumor energy and anabolic supply is the immediate cause for LDHA as a potential target. Granchi et al. (105) found that N-hydroxyindole-based inhibitors of LDHA, which are isoselective and compete with metabolic substrates, reducing the intracellular conversion of glucose to lactate. Unfortunately, studies like these on LDHA inhibitors often predate the discovery of histone lactylation. Therefore, the direct effect on histone lactylation is not discussed in this paper.

### 4.2 Inhibit the function of lactate transporter and reduce histone lactylation

Inhibitors that block the function of lactate transporters have shown some potential in tumor therapy. This is because tumor cells typically have a high rate of glycolysis, even when oxygen is plentiful. To maintain intracellular pH balance and eliminate lactate, tumor cells rely on lactate transporters to move lactate out of the cell (106). Inhibition of lactate transport may also change the pH of the TME, and the transformation of metabolic pattern leads to glucose deprivation, which indirectly reduces the proliferation of tumor cells in hypoxic areas. Therefore, inhibitors targeting lactate transporters can be used as an adjunctive method in combination with conventional chemotherapy or radiotherapy to reduce the series of adverse effects of histone lactylation. MCT is involved in the transport process and plays a key role in the uptake and release of lactate, while the transport results of lactate also affect the process of histone lactylation. Therefore, directing treatment to MCT is a promising new tumor treatment strategy (107, 108). Numerous preclinical trials of MCT blockers have identified this possibility. It has also been reported in the studies of colorectal cancer, breast cancer and cervical cancer. Synthetic drugs and natural products, such as Simvastatin and quercetin, have also demonstrated the therapeutic potential of MCT blockers in some tumors (109, 110). In fact, there are many types of MCTS, and the related drug blockers don’t block all types of MCTS as we thought. For example, in the small cell lung cancer Phase I trial conducted in the United Kingdom, the MCT blocker AZD 3965 was evaluated to show better inhibition of MCT1 (111). In fact, histone lactylation is a dynamic system of epigenetic modification processes. There is still a long way to go in the research around inhibiting the function of lactate transporters, especially in human experimental studies, which should be more cautious.

## 5 Conclusion and prospect

There’s been a lot of progress in the epigenetics of tumors over the last long time. More and more powerful computational tools and experimental studies have contributed to the explosive development of this field, providing us with a new research idea for a more comprehensive view and analysis of tumors (112, 113). The discovery of histone lactylation helps us to connect the complex relationship between tumor and immune, metabolic and microenvironment, constructing a clearer global view of tumor metabolic reprogramming. In this manuscript, we have made a comprehensive summary and discussion on the process of histone lactylation and the tumor targeted therapy around this direction. We reveal the importance and future prospects of this field, focusing on how tumor cell metabolic reprogramming reshapes the epigenetic landscape and exploring the plasticity of histone lactylation in reverse regulation of tumor metabolism.

Histone lactylation regulates a series of dangerous events such as tumor angiogenesis, metabolic reprogramming and immune escape, contributing to the occurrence and progression of tumors. Targeted studies based on histone lactylation will provide more options for the treatment of tumors in the future. In fact, as a new field of hot research, the basic and clinical research of targeted histone lactylation still has a long way to go. There are still many questions to be answered: (a) Can therapies targeting histone lactylation work synergically with other epigenetic modification phenomena to produce a greater effect than one plus one? (b) Can we find more therapeutic sites for histone lactylation, thereby increasing the specificity of therapeutic drug selection? (c) Can we find more natural products that act on histone lactylation? In the future, considerable effort will be required to answer these questions individually. Therefore, considering the crucial role of histone lactylation in the development process of diseases and the construction of corresponding treatment strategies, it has become a potential target with great application prospects in the field of disease treatment. However, it should be clearly stated that the research currently conducted around the core direction of “achieving disease treatment by regulating the level of histone lactylation” is still generally in the exploratory initial stage and has not yet formed a mature theoretical system and technical path. In particular, we need to pay particular attention to the important role that lactate plays in this process. Not only has it become an important hub to intricately link various factors that affect the initiation and development of tumors, but also the research on lactate can directly benefit clinical research, which will provide unlimited ideas and possibilities for the precision treatment of tumors.
